# Cell-free DNA levels associate with COPD exacerbations and mortality

**DOI:** 10.1186/s12931-023-02658-1

**Published:** 2024-01-18

**Authors:** Sarah A. Ware, Corrine R. Kliment, Luca Giordano, Kevin M. Redding, William L. Rumsey, Stewart Bates, Yingze Zhang, Frank C. Sciurba, S. Mehdi Nouraie, Brett A. Kaufman

**Affiliations:** 1 Department of Medicine, Division of Cardiology, Center for Metabolism and Mitochondrial Medicine, University of Pittsburgh School of Medicine, 200 Lothrop Street BST W1044, Pittsburgh, PA 15261 USA; 2 Department of Medicine, Division of Pulmonary, Allergy and Critical Care Medicine, University of Pittsburgh School of Medicine, Pittsburgh, PA USA; 3 GlaxoSmithKline Respiratory Therapeutic Area Unit, Collegeville, PA USA; 4 GlaxoSmithKline Respiratory Therapeutic Area Unit, Stevenage, UK; 5 UPMC Montefiore Hospital, NW628 3459 Fifth Avenue, Pittsburgh, PA 15213 USA

## Abstract

**The question addressed by the study:**

Good biological indicators capable of predicting chronic obstructive pulmonary disease (COPD) phenotypes and clinical trajectories are lacking. Because nuclear and mitochondrial genomes are damaged and released by cigarette smoke exposure, plasma cell-free mitochondrial and nuclear DNA (cf-mtDNA and cf-nDNA) levels could potentially integrate disease physiology and clinical phenotypes in COPD. This study aimed to determine whether plasma cf-mtDNA and cf-nDNA levels are associated with COPD disease severity, exacerbations, and mortality risk.

**Materials and methods:**

We quantified mtDNA and nDNA copy numbers in plasma from participants enrolled in the Evaluation of COPD Longitudinally to Identify Predictive Surrogate Endpoints (ECLIPSE, *n* = 2,702) study and determined associations with relevant clinical parameters.

**Results:**

Of the 2,128 participants with COPD, 65% were male and the median age was 64 (interquartile range, 59–69) years. During the baseline visit, cf-mtDNA levels positively correlated with future exacerbation rates in subjects with mild/moderate and severe disease (Global Initiative for Obstructive Lung Disease [GOLD] I/II and III, respectively) or with high eosinophil count (≥ 300). cf-nDNA positively associated with an increased mortality risk (hazard ratio, 1.33 [95% confidence interval, 1.01–1.74] per each natural log of cf-nDNA copy number). Additional analysis revealed that individuals with low cf-mtDNA and high cf-nDNA abundance further increased the mortality risk (hazard ratio, 1.62 [95% confidence interval, 1.16–2.25] per each natural log of cf-nDNA copy number).

**Answer to the question:**

Plasma cf-mtDNA and cf-nDNA, when integrated into quantitative clinical measurements, may aid in improving COPD severity and progression assessment.

**Supplementary Information:**

The online version contains supplementary material available at 10.1186/s12931-023-02658-1.

## Take-home message

In this COPD cohort, we found that elevated cf-nDNA predicts mortality while elevated cf-mtDNA predicts future exacerbations, with a hazard ratio of 1.3. Integration of both cf-mtDNA and cf-nDNA measures improved mortality predictions to 1.66 in our study.

**Supplementary Information:**

The online version contains supplementary material available at 10.1186/s12931-023-02658-1.

## Introduction

Chronic obstructive pulmonary disease (COPD) is a progressive lung disease characterized by alveolar destruction, airway remodeling, and inflammation [1, 2]. Environmental, behavioral, and genetic factors play pivotal roles in disease onset and progression. Cigarette smoke (CS) exposure is the most prominent risk factor for developing COPD [3].

CS exposure induces oxidative stress and mitochondrial dysfunction that can cause mitochondrial DNA (mtDNA) and nuclear (nDNA) damage and release into the cytosol or extracellular space [4–7]. Due to its evolution from bacterial origins, mislocalized mtDNA acts as a damage-associated molecular pattern that can promote inflammation by binding to DNA-sensing receptors [8, 9]. Indeed, cell-free mitochondrial (cf-mtDNA) and nuclear DNA (cf-nDNA) have been investigated as biomarkers of disease progression and mortality in critical illness [10] and idiopathic pulmonary fibrosis (IPF) [11, 12]. Cf-mtDNA may be mechanistically linked to pathology, as intratracheal mtDNA administration in mice triggered macrophage infiltration and proinflammatory cytokine production [13]. Mitochondrial dysfunction [5, 14] and cellular necrosis [7] are thought to contribute to COPD pathogenesis and are associated with the release of cf-mtDNA and cf-nDNA into the bloodstream and other compartments [15, 16]. Cf-mtDNA and cf-nDNA have been detected in the bronchoalveolar lavage [17, 18] and serum [6] of mice exposed to CS. Moreover, CS-induced release of DNA from neutrophils and macrophages contributed to the formation of extracellular traps. Decreasing these extracellular traps by intraperitoneal DNase-I reduced lung injury [19].

Despite the current understanding of COPD pathogenesis, there remains a lack of good biological indicators capable of predicting COPD disease phenotypes and clinical trajectories, including acute exacerbations [20–22]. The association of novel biomarkers with different COPD stages, phenotypes, and outcomes could provide insight into pathogenesis and the development of targeted therapeutics. The Rome Proposal from COPD panel experts highlighted the need to incorporate predictive biomarkers in assessing acute exacerbations in COPD [23]. A recent study reported that cf-mtDNA levels were higher in the plasma of 700 individuals with mild to moderate COPD in the SPIROMICS cohort [24]. However, these findings have not been validated in a larger, independent cohort. In this study, we tested the association of plasma cf-mtDNA and cf-nDNA levels with clinical measures of COPD severity, rate of exacerbations, and mortality in the Evaluation of COPD Longitudinally to Identify Predictive Surrogate Endpoints (ECLIPSE) cohort.

## Methods

### Study participants

We analyzed prospectively collected plasma samples from participants enrolled in the ECLIPSE cohort, a longitudinal multicenter study of non-smokers and current or former smokers with and without COPD. The study design and distribution of disease have been previously described [25, 26]. Details of the study design and clinical parameters collected are summarized in the online Supplementary Information.

### Automated cell-free DNA (cf-DNA) isolation from plasma

Total cf-DNA was isolated from plasma using a previously validated automated, high throughput methodology [27]. A detailed description of cf-DNA isolation and quantitation from plasma are provided in the online Supplementary Information.

### Quantitative polymerase chain reaction (qPCR) and digital polymerase chain reaction (dPCR)

Cf-mtDNA and cf-nDNA levels were measured simultaneously by TaqMan-based duplex qPCR as previously described [27]. The assay quantified mitochondrial-encoded human NADH:ubiquinone oxidoreductase core subunit 1 (ND1) and nuclear-encoded human beta-2-microglobulin (B2M). Serial dilutions of pooled human placenta DNA were used as a standard curve as we previously described [28]. Assay sequences (Supplementary Table S1) are provided in the online Supplementary Information.

mtDNA and nDNA copy numbers (copies/µL) of the standard curve were determined by dPCR as previously described [29] and used to calculate copy numbers for the experimental samples by linear regression. Using the QuantStudio 3D Digital PCR System and associated reagents (Thermo Fisher, Waltham, Massachusetts, USA), mtDNA and nDNA copy numbers were measured separately using singleplex ND1 and B2M assays. All reactions were performed in duplicate (two chips). Details of both qPCR and dPCR methods are provided in the Supplementary Information.

### Statistical analysis

**Fig. 1:**
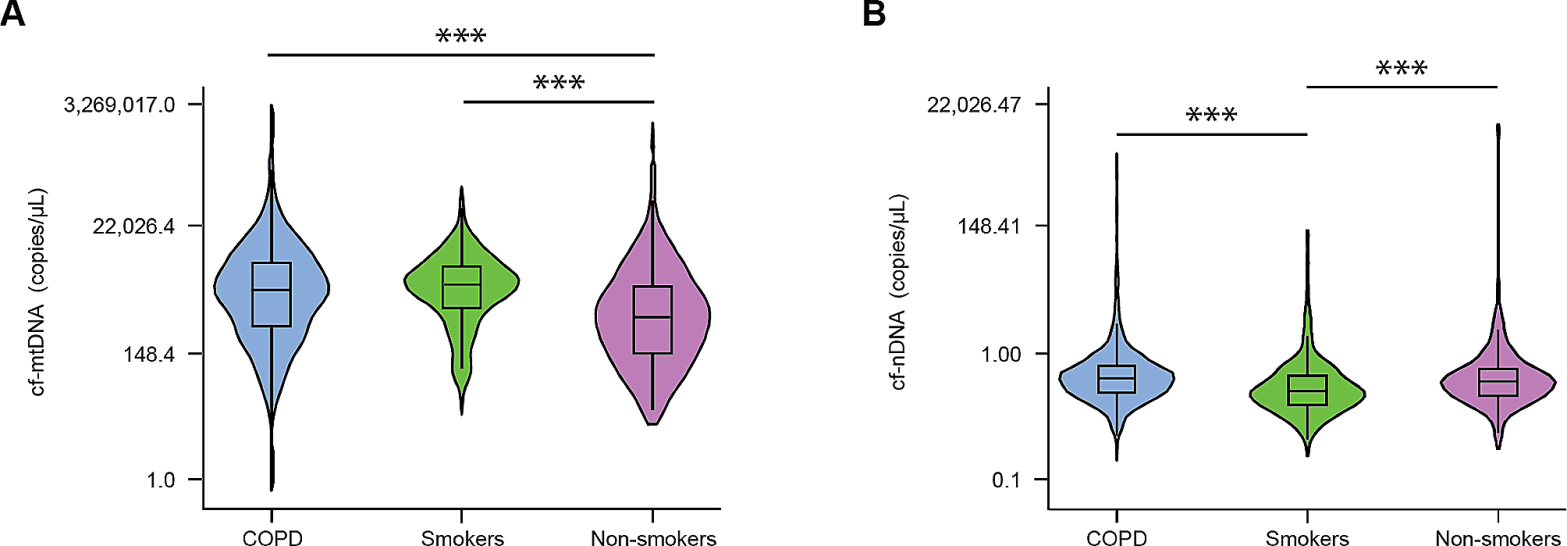
cf-mtDNA levels are higher in participants with COPD. Baseline (**A**) cf-mtDNA and (**B**) cf-nDNA levels were assessed in participants with COPD (*n* = 2,128), smokers (*n* = 331), and non-smokers (*n* = 243*)*. The results are presented as median, with the box indicating Q1 and Q3 quartiles and the whiskers indicating the range. To format the y-axis, we used the natural logarithmic scale of data and then transformed it back to nominal data for ease of interpretation. Statistical analysis was performed using ANOVA with Bonferroni post hoc pairwise comparison. ***P value < 0.001. cf-mtDNA = cell-free mitochondrial DNA; cf-nDNA = cell-free nuclear DNA; COPD = chronic obstructive pulmonary disease

**Fig. 2:**
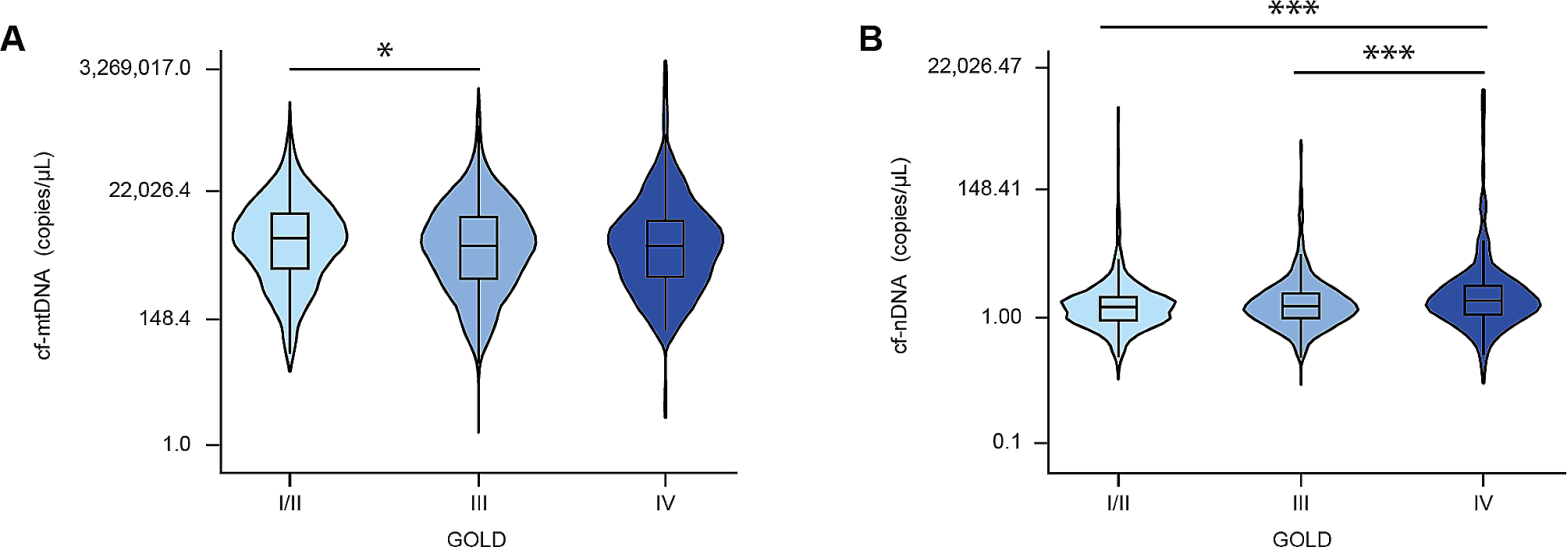
cf-mtDNA levels are higher in participants classified as GOLD stage I/II while cf-nDNA levels are higher in participants classified as GOLD stage IV. Baseline (**A**) cf-mtDNA and (**B**) cf-nDNA levels were assessed in participants with COPD who were classified as GOLD stage I/II (*n* = 944), III (*n* = 893), or IV (*n* = 290). The results are presented as median, with the box indicating Q1 and Q3 quartiles and the whiskers indicating range. To format the y-axis, we used the natural logarithmic scale of data and then transformed it back to nominal data for ease of interpretation. Statistical analysis was performed using ANOVA with Bonferroni post hoc pairwise comparison. *P value < 0.05, ***P value < 0.001. cf-mtDNA = cell-free mitochondrial DNA; cf-nDNA = cell-free nuclear DNA; COPD = chronic obstructive pulmonary disease; GOLD = Global Initiative for Obstructive Lung Disease

**Fig. 3:**
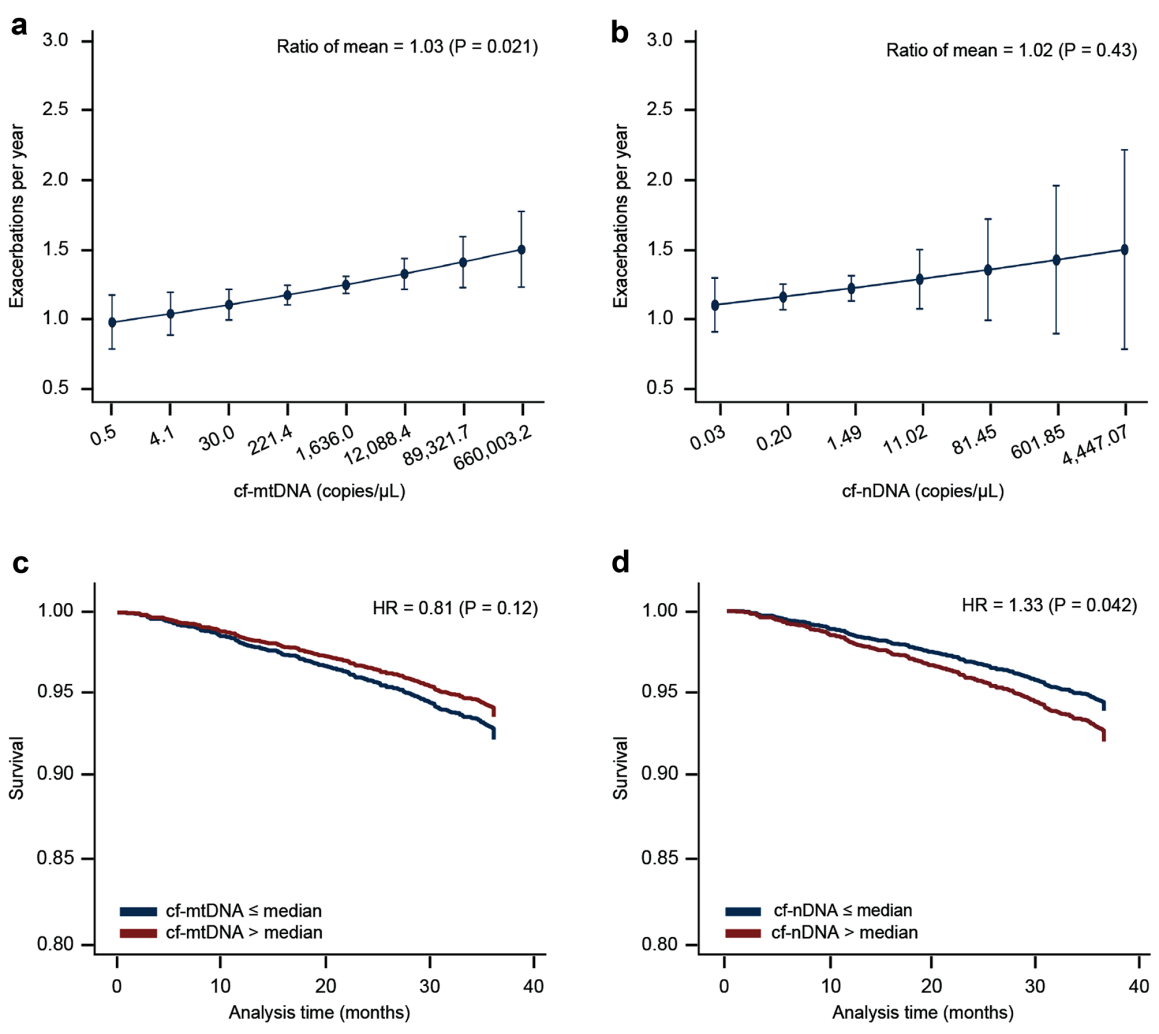
Higher cf-mtDNA levels at baseline are associated with an increased rate of yearly exacerbations while cf-nDNA levels are associated with an increased risk of mortality in participants with COPD. Baseline (**A**) cf-mtDNA and (**B**) cf-nDNA levels were evaluated for their association with the average number of exacerbations per year during the 3-year study period in all participants with COPD (*n* = 2,106). The results are presented as adjusted mean with 95% confidence intervals. Statistical analysis was performed using gamma regression after adjusting for age, sex, and body mass index. In (**A**) and (**B**), we used the natural logarithmic scale of data and then transformed back the x-axis to nominal data for ease of interpretation. Baseline (**C**) cf-mtDNA and (**D**) cf-nDNA levels were evaluated for their association with the risk of mortality in all participants with COPD (n = 2,126). Statistical analysis was performed using Cox proportional hazard regression. cf-mtDNA = cell-free mitochondrial DNA; cf-nDNA = cell-free nuclear DNA; COPD = chronic obstructive pulmonary disease; HR = hazard ratio

All analyses regarding the ECLIPSE cohort were performed using Stata version 16.0 (StataCorp). Continuous variables were presented as the median (interquartile range [IQR]) in three study subject groups and tested using ANOVA. We used natural logarithmic transformation of cf-mtDNA and cf-nDNA to optimally transform the data to a normal distribution for statistical comparison. In Figs. 1–3, cf-DNA levels were transformed back to nominal data (copies/µL) for ease of interpretation. Categorical variables were presented as frequencies and tested using Fisher’s exact test.

We applied linear regression analysis to calculate the unadjusted and adjusted (for sex, body mass index, and age) standardized regression (correlation) coefficient of continuous outcomes with cf-mtDNA and cf-nDNA levels (with natural log transformation). For binary outcomes, we used logistic regression analysis. We noted that the rate of exacerbation was positively skewed (mean = 1.2, SD = 1.4, P < 0.001 against a normal distribution). Therefore, we used the generalized linear model with gamma family distribution with a log link and robust variance estimation to analyze the association between cf-mtDNA and cf-nDNA levels and the rate of exacerbations. This model is appropriate for non-negative data with heteroscedasticity. Time to mortality was calculated using the Cox proportional regression analysis. Hazard ratio (95% confidence interval [CI]) was reported for high cf-mtDNA and cf-nDNA using a median level in all COPD patients. The assumption of Cox analysis was assessed and verified. We did not adjust the P values for multiple hypothesis testing. We did not adjust for missing outcomes because the effects were negligible in most cases.

## Results

### Characteristics of the tested cohort

The ECLIPSE cohort subgroups and their clinical characteristics are shown in Supplementary Figure S1 and Table 1, respectively. From the 3,186 samples provided, 484 were excluded due to insufficient plasma volume. The remaining 2,702 subjects were separated into 243 non-smokers, 331 current or former smokers without airflow obstruction, or 2,128 current or former smokers with COPD.

The ECLIPSE participants were recruited from United States of America, Canada, and Europe. The subjects were primarily white (97–98%) (Table 1), preventing the detection of socio-racial contributions. Compared to non-smokers and smokers, individuals with COPD were predominantly older males with a lower body mass index (BMI), which was adjusted for in subsequent analyses. Each participant with COPD was assigned a Global Initiative for Obstructive Lung Disease (GOLD) stage based on the severity of their airway limitation [30]. One subject was GOLD stage I (0.1%, mild disease), 943 individuals were stage II (44%, moderate disease), 893 were stage III (42%, severe disease), and 290 were stage IV (14%, very severe disease) (Table 1, Supplementary Figure S1). GOLD stages I and II were grouped for statistical analysis. At baseline, the median FEV_1_% of the COPD group was 47% (interquartile range [IQR], 36–61%) (Table 1). As expected, packs per year, FEV_1_% [31, 32], FVC% [31, 32], FEV_1_/FVC [31, 32], eosinophil blood counts [33], St. George’s Respiratory Questionnaire (SGRQ) total score [31], and emphysema were significantly different in the COPD group compared to both control groups (P < 0.001).

**Table 1:** Baseline characteristics and cell-free DNA levels of study participants

	COPD *n* = 2,052 − 2,128	Smokers without airflow obstruction *n* = 298–331	Non-smokers *n* = 181–243	P value^A^
Age, median (IQR)	64 (59–69)	55 (48–62)	54 (47–61)	< 0.001
Male, *n* (%)	1,390 (65)	183 (55)	92 (38)	< 0.001
BMI, median (IQR)	26.0 (22.6–29.5)	26.3 (23.6–29.1)	26.6 (23.9–30.3)	0.008
White, *n* (%)	2,081 (98)	322 (97)	237 (98)	0.65
Current non-smoker, *n* (%)	744 (36)	189 (58)	--	< 0.001
Packs per year, median (IQR)	44 (30–60)	28 (18–39)	--	< 0.001
Post-bronchodilator FEV_1_ (%), median (IQR)	47 (36–61)	108 (100–117)	116 (103–123)	< 0.001
FVC (%), median (IQR)	80 (65–93)	112 (103–121)	118 (107–127)	< 0.001
Post-bronchodilator FEV_1_/FVC, median (IQR)	58 (48–70)	101 (97–106)	104 (100–108)	< 0.001
cf-mtDNA (copies/µL), median (IQR)	1,042 (262-3,266)	1,381 (563-2,933)	367 (91 − 1,299)	< 0.001
cf-nDNA (copies/µL), median (IQR)	0.54 (0.31–0.92)	0.34 (0.20–0.61)	0.49 (0.29–0.80)	< 0.001
Eosinophil count (cells/µL), median (IQR)	180 (110–280)	170 (100–240)	130 (80–210)	< 0.001
GOLD stage, *n* (%)				
I	1 (0.1)	--	--	NA
II	943 (44)			
III	893 (42)			
IV	290 (14)			
SGRQ total score, median (IQR)	51 (35–66)	5 (3–11)	2 (0–6)	< 0.001
BODE index, median (IQR)	3 (2–5)	--	--	NA
Borg scale, median (IQR)*	4 (2–5)	--	--	NA
Emphysema presence, *n* (%)	747 (39)	17 (5)	0 (0)	< 0.001
6MWD (m), median (IQR)	370 (300–447)	--	--	NA
No. of prior exacerbations (%)				
0	1,121 (53)	--	--	NA
1	540 (25)			
2	266 (13)			
3+	201 (9)			

*Definition of abbreviations*: 6MWD = 6-minute walk distance; BMI = body mass index; BODE = body mass index, airflow obstruction, dyspnea, and exercise; cf-mtDNA = cell-free mitochondrial DNA; cf-nDNA = nuclear DNA; COPD = chronic obstructive pulmonary disease; ECLIPSE = Evaluation of COPD Longitudinally to Identify Predictive Surrogate Endpoints; FEV_1_ = forced expiratory volume in one second; FVC = forced vital capacity; GOLD = Global Initiative for Obstructive Lung Disease; IQR = interquartile range; SGRQ = St. George’s Respiratory Questionnaire

^A^From t-test for quantitative data and Fisher’s exact test for categorical data

*n = 935

### cf-mtDNA and cf-nDNA copy number in plasma

To determine if either cf-mtDNA or cf-nDNA levels in the plasma were significantly different between subgroups, cf-DNA was isolated in a blinded fashion from plasma samples collected during the baseline visit. Then, cf-mtDNA and cf-nDNA levels were determined by quantitative PCR followed by interpolation to a standard curve. Digital PCR (dPCR) was used to determine the copies/µL of the standard curve. Copies/µL of the experimental samples were interpolated from the standard curve by linear regression calculations. After adjusting for age, sex, and BMI, cf-mtDNA levels were significantly higher in the plasma of participants with COPD and smokers than non-smokers (P < 0.001, Fig. 1A). There was no difference in cf-mtDNA copy levels between COPD subjects and smokers. The median cf-mtDNA level was 1,042 copies/µL (IQR, 262-3,266 copies/µL) for COPD participants, 1,381 copies/µL (IQR, 563-2,933 copies/µL) for smokers, and 367 copies/µL (IQR, 91 − 1,299 copies/µL) for non-smokers (Table 1).

cf-nDNA copies were significantly higher in subjects with COPD or non-smokers than smokers (P < 0.001, Fig. 1B). In contrast, there was no difference in median cf-nDNA copy levels between individuals with COPD and non-smokers. The median cf-nDNA level was 0.54 copies/µL (IQR, 0.31–0.92 copies/µL) for COPD participants, 0.34 copies/µL (IQR, 0.20–0.61 copies/µL) for smokers, and 0.49 copies/µL (IQR, 0.29–0.80 copies/µL) for non-smokers (Table 1).

### Associations between cf-mtDNA and cf-nDNA copies in plasma and clinical variables

To determine if COPD severity was associated with higher cf-DNA levels in this cohort, the participants were stratified by GOLD stage. The median cf-mtDNA level for mild/moderate (GOLD I/II) COPD cases was 1,220 copies/µL (IQR, 334-3,639), 911 copies/µL (IQR, 219-3,151) for severe (GOLD III), and 901 copies/µL (IQR, 244-2,689) for very severe (GOLD IV) airway obstruction (Table 2). cf-mtDNA levels were significantly increased in GOLD I/II cases compared to GOLD III (P < 0.05, Fig. 2A). There was no statistically significant difference in cf-mtDNA levels between GOLD I/II and GOLD IV groups or between GOLD III and GOLD IV groups. The median cf-nDNA level for GOLD I/II cases was 0.5 copies/µL (IQR, 0.3–0.8), 0.5 copies/µL (IQR, 0.3–0.9) for GOLD III, and 0.7 copies/µL (IQR, 0.4–1.3) for GOLD IV (Table 2). In contrast, cf-nDNA levels were significantly increased in samples collected from GOLD IV cases compared to either GOLD I/II or III cases (P values < 0.001, Fig. 2B). There was no difference between the levels of cf-nDNA in GOLD I/II and III groups.

**Table 2:** Baseline cell-free DNA Levels by GOLD Stage

	GOLD I/II *n* = 944	GOLD III *n* = 893	GOLD IV *n* = 290	P value^A^
cf-mtDNA (copies/µL), median (IQR)	1,220 (334-3,639)	911 (219-3,151)	901 (244-2,689)	0.017
cf-nDNA (copies/µL), median (IQR)	0.5 (0.3–0.8)	0.5 (0.3–0.9)	0.7 (0.4–1.3)	< 0.001

*Definition of abbreviations*: GOLD = Global Initiative for Obstructive Lung Disease; cf-mtDNA = cell-free mitochondrial DNA; cf-nDNA = cell-free nuclear DNA. ^A^From t-test for quantitative data and Fisher’s exact test for categorical data

We then sought to determine if either cf-mtDNA or cf-nDNA levels were associated with other clinical variables in the COPD group. The correlations (both unadjusted and adjusted) are presented in Table 3. The adjustment for age, sex, and BMI did not influence the associations for either DNA measure. cf-mtDNA levels were not associated with FEV_1_%, FEV_1_/FVC, the body mass index, airflow obstruction, dyspnea, and exercise (BODE) index, or SGRQ total score (Table 3). However, cf-mtDNA levels were positively correlated with FVC% (r = 0.05, P = 0.026), the Borg exertional dyspnea scale (r = 0.18, P < 0.001), and the number of prior exacerbations that occurred the year before the first study visit (r = 0.05, P = 0.031) and negatively correlated with GOLD stage (r = -0.06, P = 0.009) and 6-minute walk distance (6MWD) (r = -0.04, P = 0.035). cf-nDNA levels were negatively correlated with FEV_1_% (r = -0.13, P < 0.001), FVC% (r = -0.10, P < 0.001), FEV_1_/FVC (r = -0.10, P < 0.001), and the 6MWD (r = -0.13, P < 0.001). cf-nDNA levels were positively correlated with GOLD stage (r = 0.13, P < 0.001), the BODE index (r = 0.15, P < 0.001), SGRQ total score (r = 0.12, P < 0.001), Borg scale (r = 0.07, P = 0.029), and the number of prior exacerbations (r = 0.06, P = 0.005). Neither cf-mtDNA nor cf-nDNA levels were associated with the presence of emphysema.

**Table 3:** Correlation (P value) between baseline DNA measures and clinical parameters in participants with COPD

	cf-mtDNA copy number (natural log)		cf-nDNA copy number (natural log)	
	Unadjusted	Adjusted^†^	Unadjusted	Adjusted^†^
Post-bronchodilator FEV_1_%	0.03 (0.14)	0.04 (0.07)	-0.12 (< 0.001)	-0.13 (< 0.001)
FVC%	0.05 (0.028)	0.05 (0.026)	-0.11 (< 0.001)	-0.10 (< 0.001)
Post-bronchodilator FEV_1_/FVC	-0.02 (0.26)	-0.01 (0.56)	-0.07 (0.001)	-0.10 (< 0.001)
GOLD stage	-0.05 (0.025)	-0.06 (0.009)	012 (< 0.001)	0.13 (< 0.001)
BODE index	0.00 (0.99)	0.00 (0.95)	0.15 (< 0.001)	0.15 (< 0.001)
SGRQ total score	0.04 (0.07)	0.04 (0.09)	0.12 (< 0.001)	0.12 (< 0.001)
Borg scale^‡^	0.18 (< 0.001)	0.18 (< 0.001)	0.06 (0.03)	0.07 (0.029)
6MWD	-0.03 (0.14)	-0.04 (0.035)	-0.15 (< 0.001)	-0.13 (< 0.001)
Presence of emphysema^§^	1.05 (0.06)	1.05 (0.08)	0.93 (0.13)	0.94 (0.20)
No. of prior exacerbations	0.05 (0.016)	0.05 (0.031)	0.06 (0.012)	0.06 (0.005)

*Definition of abbreviations*: 6MWD = 6-minute walk distance; BODE = body mass index, airflow obstruction, dyspnea, and exercise; cf-mtDNA = cell-free mitochondrial DNA; cf-nDNA = cell-free nuclear DNA; FVC = forced vital capacity; FEV_1_ = forced expiratory volume in one second; GOLD = Global Initiative for Obstructive Lung Disease; SGRQ = St. George’s Respiratory Questionnaire

^†^Adjusted for age, body mass index, and sex

^‡^*n* = 940

^§^Odds ratio (P value)

### Associations between cf-mtDNA copies, rate of future exacerbations, and eosinophil count

COPD exacerbations were defined as a clinical episode of worsening pulmonary symptoms requiring systemic corticosteroids, antibiotics, or both [34]. The average rate of exacerbations over the three-year study period was provided in the clinical data. After adjusting for age, sex, and BMI, regression analysis showed that higher levels of cf-mtDNA were associated with an increased rate of exacerbations for the 3-year study (Fig. 3A). The ratio of the mean from the gamma regression was 1.03, indicating a 3% increase in exacerbations per each natural log increase of mtDNA (P = 0.021). A similar result arose from unadjusted data (ratio of the mean, 1.03, P = 0.030, data not shown). An association with exacerbation rate was not observed for cf-nDNA levels (Fig. 3B).

We next examined the association between each cf-DNA measure and the rate of exacerbations by GOLD stage (Table 4). Participants classified as GOLD I/II or GOLD III with higher cf-mtDNA levels experienced more exacerbations in the time between visits (ratio of the mean, 1.06, P = 0.011 for GOLD I/II and ratio of the mean, 1.05, P value = 0.012 for GOLD III). However, this association was not present in participants classified as GOLD IV. No associations were observed for cf-nDNA levels.

**Table 4:** Effects of DNA measures on the rate of exacerbations by GOLD stage^†^

	GOLD I/II *n* = 933	GOLD III *n* = 886	GOLD IV *n* = 286
cf-mtDNA copy number (natural log)	1.06 (0.011)	1.05 (0.012)	0.99 (0.75)
cf-nDNA copy number (natural log)	1.01 (0.84)	0.95 (0.19)	0.98 (0.66)

*Definition of abbreviations*: GOLD = Global Initiative for Obstructive Lung Disease; cf-mtDNA = cell-free mitochondrial DNA; cf-nDNA = cell-free nuclear DNA

^†^Cells represent the ratio of the mean (P value) from a gamma regression. All analyses are adjusted for age, body mass index, and sex

Using established thresholds [35], 1,640 COPD participants had normal eosinophil levels (< 300 cells/µL) and 430 had elevated levels (≥ 300 cells/µL) at the baseline visit. COPD participants with higher levels of cf-mtDNA and a high eosinophil count experienced an increased rate of exacerbations (ratio of the mean, 1.09, P = 0.007) (Table 5). This trend was not observed in COPD participants with a low eosinophil count. No association was observed for cf-nDNA levels.

**Table 5:** Effects of DNA measures on the rate of exacerbations by eosinophil count^†^

	Eosinophil count < 300 *n* = 1,618 − 28*	Eosinophil count ≥ 300 *n* = 423-6*
cf-mtDNA copy number (natural log)	1.02 (0.25)	1.09 (0.007)
cf-nDNA copy number (natural log)	1.02 (0.60)	1.07 (0.21)

*Definition of abbreviations*: cf-mtDNA = cell-free mitochondrial DNA; cf-nDNA = cell-free nuclear DNA

^†^Cells represent the ratio of the mean (P value) from a gamma regression. All analyses are adjusted for age, body mass index, and sex. *values are different depending on DNA measure

### Association between cf-nDNA copies and mortality

All-cause mortality was documented up to day 1,060 of the study. The cause of death was not further investigated [34]. In adjusted analyses, we observed no association between cf-mtDNA levels and mortality risk (Fig. 3C). In contrast, there was a significant association between higher cf-nDNA levels and decreased survival (hazard ratio [HR], 1.33, 95% confidence interval [CI], 1.01–1.74, P = 0.042) (Fig. 3D), indicating that there was 33% increase in mortality for each natural log increase of cf-nDNA.

### Combined analysis of relative cf-mtDNA and cf-nDNA copies enhances survival prediction

**Fig. 4:**
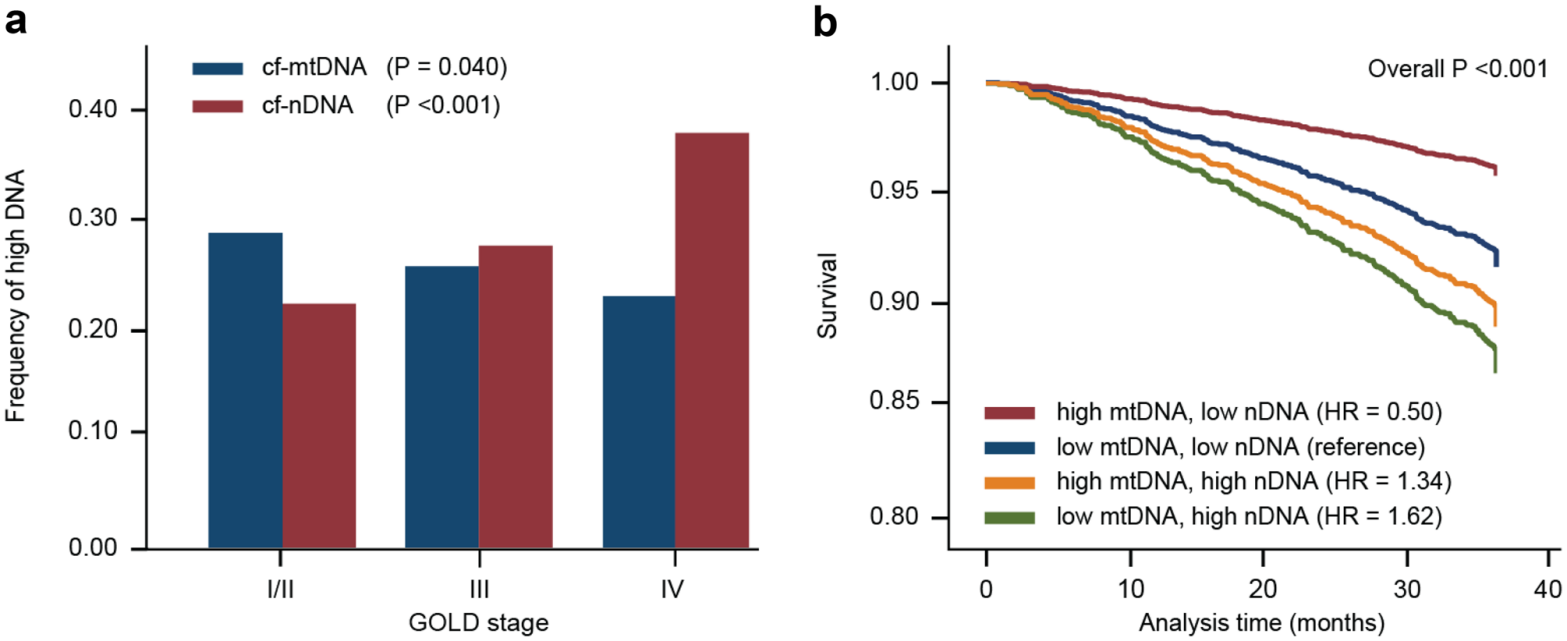
Combined measures of low cf-mtDNA and high cf-nDNA levels are associated with increased mortality risk. (**A**) Samples across the COPD population were binned into quartiles. The frequency of the top quartile of cf-mtDNA and cf-nDNA was determined across GOLD stages. Statistical analysis was performed using test for linear trend. (**B**) Low and high quartiles of cf-mtDNA and cf-nDNA were combined, demonstrating an increase in the strength of the association with mortality. cf-mtDNA = cell-free mitochondrial DNA; cf-nDNA = cell-free nuclear DNA; GOLD = Global Initiative for Obstructive Lung Disease; HR = hazard ratio

Because cf-mtDNA and cf-nDNA demonstrated a reciprocal relationship regarding COPD severity (Table 2; Fig. 2) and are associated with distinct clinical parameters (exacerbation rate and mortality, respectively), we wanted to determine whether the combination of these measures would improve the prediction of survival. We established quartile assignments for cf-mtDNA and cf-nDNA levels for all participant samples and calculated the frequency of being in the highest quartile for each GOLD stage (Fig. 4A). The frequency of samples being in the top quartile for cf-nDNA increased as disease stage increased (P < 0.001), while the frequency for cf-mtDNA decreased (P = 0.040). Thus, having high cf-mtDNA and low cf-nDNA was a common feature of GOLD stage I/II, whereas having low cf-mtDNA and high cf-nDNA was a common feature of GOLD stage IV. Using low cf-mtDNA and low cf-nDNA as a neutral reference group, we calculated the survival HR for the other combinations (Fig. 4B). The HR for the high cf-mtDNA and low cf-nDNA group was 0.5 (95% CI, 0.29–0.84, overall P < 0.001), indicating that participants with this characteristic had twice the survival rate compared to the neutral reference group. Notably, the low cf-mtDNA and high cf-nDNA group showed worse outcomes, with a HR of 1.62 (95% CI, 1.16–2.25, overall P < 0.001).

## Discussion

Quantifiable indicators that associate with COPD pathogenesis are needed to classify endotypes, predict outcomes, and assess treatment responsiveness. The incorporation of new biomarkers for the prediction of acute exacerbations was highlighted in the Rome Proposal by a panel of COPD experts [23]. The ability to assess the trajectories of COPD endotypes will involve recognizing associated biomarkers. Several attempts to categorize patients based on clinical and radiologic parameters have been made; however, there remains a missing link between such parameters and underlying disease mechanisms [36, 37].

In a subset of the SPIROMICS cohort, plasma cf-mtDNA was elevated in patients with mild to moderate COPD compared to severe COPD [24]. Initially, mtDNA was associated with exacerbations and clinical outcomes (6MWD, SGRQ total score, or COPD Assessment Test score) but was no longer significant after adjusting for loss of follow-up data. In the same cohort, higher urine mtDNA was associated with increased symptoms and worse exercise tolerance [31]. In a separate, larger cohort, our study validates the finding that cf-mtDNA was higher in the plasma of patients with mild to moderate COPD. Moreover, our study showed that elevated cf-mtDNA was positively associated with FVC%, the Borg exertional dyspnea scale, and number of prior exacerbations and negatively associated with GOLD stage and 6MWD. Furthermore, cf-mtDNA predicted future exacerbations, particularly in GOLD I-III participants or those with high eosinophil counts, suggesting its potential utility as a stratifying marker of at-risk subgroups for exacerbations. Cf-mtDNA has proinflammatory implications [13] and therefore may promote chronic inflammatory responses and exacerbations in COPD.

Neither study involving the SPIROMICS cohort reported cf-nDNA levels. In the present study, we showed that cf-nDNA was negatively associated with FEV_1_%, FVC%, FEV_1_/FVC, and 6MWD and positively associated with GOLD stage, BODE index, SGRQ total score, the Borg scale, number of prior exacerbations, and an increased risk for mortality. While cf-mtDNA is predictive of exacerbations, we note that it is not associated with increased mortality risk and the cf-mtDNA data seemingly separate exacerbations from survival. Integrating both cf-mtDNA and cf-nDNA measures improved mortality predictions in our study. Specifically, participants with low cf-mtDNA and high cf-nDNA levels showed worse outcomes among patients with COPD. As such, multi-factor assessments are likely necessary to refine risk prediction. The distinct predictive nature of integrated cf-mtDNA and cf-nDNA measurements should be investigated in additional COPD patient cohorts stratified by endotypes, such as early age-onset COPD, those with rapid decline in lung function, or frequent exacerbations.

cf-nDNA is passively released during cell death processes such as apoptosis or necrosis [15, 38]. In contrast, cf-mtDNA may result from active release mechanisms, including microvesicles [6, 39]. Cardiomyocytes release mitochondria-containing extracellular vesicles (EV) in response to stimulation with isoproterenol [40]. Therefore, some types of EV release may represent a compensatory mechanism in response to acute cellular damage that overwhelms the normal degradation pathways. Such damage leads to the redirection of endomembrane lysosomal trafficking to secretory pathways, where resident macrophages digest the contents of EVs. We suggest that the high levels of plasma cf-mtDNA in COPD may represent a compensatory response to smoke exposure, whereby systemic cellular damage triggers microparticle release. This cellular adaptation could prevent cell death, characterized by low cf-nDNA release in the plasma, as observed in this study. Consistent with this idea, the high cf-mtDNA and low cf-nDNA group had an HR of 0.5 relative to the reference group, suggesting a significant benefit from the potentially compensatory state. In contrast, low cf-mtDNA would indicate poor adaptation to stress with high cf-nDNA levels indicating increased cell death. The low cf-mtDNA/high cf-nDNA group had an HR of 1.62, which poses a significantly higher lethality than other groups. We acknowledge that this is a hypothetical scenario and may not represent the response of all cell types or cell-free DNA production mechanisms.

In summary, higher cf-mtDNA levels were associated with future exacerbations, while higher cf-nDNA levels were associated with increased mortality risk in participants with COPD. Future studies should evaluate changes in both cell-free genomes in the context of active exacerbations or specific treatments.

### Electronic supplementary material

Below is the link to the electronic supplementary material.

**Supplementary Material 1:** Supplementary Methods

## Data Availability

The clinical data used for this study can be accessed through dbGaP Study Accession: phs001252.v1.p1. While the data are not publicly available, interested parties can apply to the pertinent data providers for data access requests.
