# Supplementary material for: Cell-free DNA levels associate with COPD exacerbations and mortality
Source: Respir Res. 2024 Jan 18;25:42. doi: 10.1186/s12931-023-02658-1 (PMC10797855; doi:10.1186/s12931-023-02658-1)
Supplement: Supplementary file 1 — Supplementary Material 1: Supplementary Methods [file 12931_2023_2658_MOESM1_ESM.docx]

**SUPPLEMENTARY INFORMATION**

**SUPPLEMENTARY METHODS**

*Study Participants*

What follows is a brief summary of the original clinical study used in the current study [[1]](https://sciwheel.com/work/citation?ids=2587675&pre=&suf=&sa=0&dbf=0). Male and female participants aged 40-75 years were recruited. Groups were defined by post-bronchodilator forced expiratory volume in 1 second (FEV_1_%), FEV_1_/forced vital capacity (FVC) ratio, and smoking history. Both non-smokers and smokers had a baseline post-bronchodilator FEV_1_% of >85% of the predicted value and FEV_1_/FVC of >0.7. Non-smokers were defined as smoking <1 pack/year. The smoker subgroup consisted of both current and former smokers without airway obstruction that had a history of smoking ≥10 packs/year. COPD participants had a baseline post-bronchodilator FEV_1_ of <80% of the predicted value, baseline post-bronchodilator FEV_1_/FVC of ≤0.7, and a smoking history of ≥10 packs per year. The study was conducted at 46 centres in 12 countries across the United States of America, Canada, and Europe.

Clinical data were collected at baseline and follow-up visits (3 months, 6 months, and then every 6 months for 3 years). These data included FEV_1_%, FVC%, and FEV_1_/FVC, the Global Initiative for Obstructive Lung Disease (GOLD) stage, body mass index, airflow obstruction, dyspnea and exercise (BODE) index, COPD-specific St. George’s Respiratory Questionnaire (SGRQ) total score, Borg scale, 6-minute walk distance (6MWD), emphysema extent based on CT scan analysis, number of exacerbations that occurred in the year before the first visit, the average number of exacerbations per year that occurred during the 3-year follow up, eosinophil count, and mortality. Borg dyspnea scores were reported at the end of the 6-minute walk. The clinical power calculation and the characteristics of the control groups were previously described [[1]](https://sciwheel.com/work/citation?ids=2587675&pre=&suf=&sa=0&dbf=0). A total of 22 participants did not have exacerbation data.

The present study only quantified cf-mtDNA and cf-nDNA levels in the baseline plasma samples and associated them with baseline clinical parameters, mortality, and yearly exacerbation rates over the 3-year study period. ECLIPSE complied with the Declaration of Helsinki and Good Clinical Practice Guidelines and was approved by the ethics committees at all participating centres. All participants provided written informed consent.

*Automated cf-DNA Isolation from Plasma*

Blood collection and plasma processing has been previously published [[2]](https://sciwheel.com/work/citation?ids=7382564&pre=&suf=&sa=0&dbf=0). Briefly, venous blood was collected into an EDTA-coated vacutainer tube on the morning after an overnight fast. Plasma was obtained by centrifugation at 2,000 x *g* for 10-15 min and stored at -80°C until analysis.

Total cf-DNA was isolated from plasma using an optimized, high-throughput methodology that has been previously described in detail [[3]](https://sciwheel.com/work/citation?ids=9722382&pre=&suf=&sa=0&dbf=0). We developed the method to reduce inter-well variation. It was validated against a manual ethanol-precipitation-based method. In the present study, sample processing was conducted in an operator-blinded fashion. Briefly, plasma was thawed and centrifuged for 10 seconds to ensure liquid was settled to the bottom of the tube. 75 µL of plasma was transferred from the provided tube into a 96 deep well plate (Thermo Fisher, Waltham, Massachusetts, USA) by a Freedom EVO 150 automated liquid handler (Tecan, Männedorf, Switzerland), which automated most of the liquid distribution. To begin digestion, 5.7 µL of 20 mg/mL Proteinase K (Thermo Fisher) and 7.5 µL of 20% sodium dodecyl sulfate (Boston BioProducts, Ashland, Massachusetts, USA) were manually dispensed to the side of each well using a HandyStep digital repeater pipette (BrandTech Scientific, Inc., Essex, CT, USA). Each plate with sealed with an adhesive PCR seal (Thermo Fisher Scientific) and covered with generic packaging tape as a secondary seal. The plates were centrifuged at 500 x *g* for 1 minute (min) and then placed in an Innova 44 Incubator Shaker (New Brunswick Scientific, Edison, NJ, USA) to incubate for 16 hours at 70°C without agitation. On the following morning, the plates equilibrated to room temperature for 15 min and were centrifuged at 500 x *g* for 1 min before removing the adhesive seals. Per well, 125 µL of MagMAX cell-free DNA lysis/binding solution (Thermo Fisher) and 5 µL of Dynabeads MyOne Silane magnetic beads (Thermo Fisher) were premixed and then dispensed using a repeater pipette to minimize bubble formation. The plates were loaded onto a KingFisher Presto (Thermo Fisher) magnetic particle processor to mix the samples. The DNA-bound magnetic beads were washed three times. The first wash was 265 µL of MagMAX cell-free DNA wash solution, followed by two washes with 475 and 200 µL of 80% ethanol. The beads were allowed to air-dry above the well for 2 min to remove any residual ethanol. To elute the DNA, the magnetic beads were mixed with 60 µL of MagMAX cell-free DNA elution solution (Thermo Fisher). Beads were captured by the Presto magnetic comb, then the elution plate was placed on a Alpaqua magnetic plate (Beverly, MA) for secondary bead removal. The eluted DNA was transferred by the liquid handler to a full skirted 96-well PCR plate (Thermo Fisher) for storage at -20°C.

*Quantitative Polymerase Chain Reaction (qPCR)*

cf-mtDNA and cf-nDNA levels were measured simultaneously by TaqMan-based duplex qPCR reactions. The assay quantified mitochondrial-encoded human NADH:ubiquinone oxidoreductase core subunit 1 (ND1) and nuclear-encoded human beta-2-microglobulin (B2M). This assay was previously validated and confirmed to give similar results to other probe sets [[4]](https://sciwheel.com/work/citation?ids=6893802&pre=&suf=&sa=0&dbf=0). For each gene, the assay was comprised of two primers and a fluorescent probe that were combined into a 20X working solution. The primer/probe ratios for the ND1 assay were 1:1 (i.e., primer-limited) and 3:1 for the nDNA assay (Integrated DNA Technologies, Newark, New Jersey, USA). The qPCR master mix used in this study was the 2X Luna Universal qPCR Master Mix (New England Biolabs, Ipswich, Maine, United States of America).

At analysis, the 96-well DNA sample plates were briefly spun to collect contents to the bottom of the plate. Each duplex reaction contained 4 µL of the qPCR master mix, 0.4 µL of each gene assay, and 3.2 µL of template DNA for a final volume of 8 µL, with each reaction performed in triplicate. The liquid handler dispensed 4.8 µL of the premixed master mix plus gene assay into a 384-well plate (Thermo Fisher), followed by 3.2 µL of template DNA. One 384-well plate (Thermo Fisher) was used for each DNA plate and amplification was performed in a QuantStudio 5 real-time PCR system (Thermo Fisher). The thermocycling conditions were as follows: 95°C for 20 sec followed by 40 cycles of 95°C for 1 sec, 63°C for 20 sec, and 60°C for 20 sec. Primer and probe sequences are summarized in supplementary table S1. A standard curve of pooled genomic DNA samples isolated from human placenta was applied to each plate. The absolute copy number for the target sequences were established by digital PCR, whose values were applied to the standard curve to interpolate the absolute copy number of the experimental samples.

Digital PCR (dPCR)

Absolute mtDNA and nDNA copy numbers (copies/µL) of the pooled standard curve samples were determined by dPCR. Using the QuantStudio 3D Digital PCR System and associated reagents (Thermo Fisher), mtDNA and nDNA copy numbers were measured separately using singleplex ND1 and B2M assays. A no-template control was included but was multiplexed (combined ND1 and B2M assays). The dPCR was prepared as previously described [[5]](https://sciwheel.com/work/citation?ids=7380525&pre=&suf=&sa=0&dbf=0). Briefly, we performed dPCR at dilutions for each probe set to yield 25-75% well vacancy in the qualified wells of the 20,000-well chip. All reactions were performed within 24 hours of preparing the standard curve and performed in duplicate chips. The reaction loaded onto each chip contained 7.25 µL of the QuantStudio 3D Digital PCR Master Mix v2 (2X), 0.73 µL of the 20X gene assay, 5 µL of DNA, and 1.52 µL water for a total volume of 14.5 µL. After loading the chips according to the manufacturer’s instructions, the PCR was performed on an Applied Biosystems ProFlex PCR system (Thermo Fisher) with QuantStudio 3D Digital PCR Chip Adapter and incline support (Thermo Fisher). The thermocycling conditions were as follows: 96°C for 10 sec, 39 cycles of 60°C for 2 min and 98°C for 30 sec, 60°C for 2 min, and 10°C indefinitely. After the PCR finished, the chips were read by a QuantStudio 3D instrument (Thermo Fisher) and the results were analyzed in the QuantStudio 3D Analysis Suite online software (Thermo Fisher). The average of the two chips was used to calculate the copies/reaction of the remaining standard curve samples. The standard curve in copies/reaction was then used to calculate the copies/reaction of the experimental samples. To calculate cf-mtDNA or cf-nDNA copies/µL of plasma, the results were adjusted to account for the volume of DNA template used in each reaction (3.2 µL), the elution volume (60 µL), and the volume of plasma from which the DNA was extracted (75 µL). Because the pooled DNA used to make the standard curve was identical for all plates, the copy number was determined once and applied uniformly for all qPCR plates.

**SUPPLEMENTARY TABLES**

**Table S1.** Sequences of Primers and Probes

|  | **Human ND1** | **Human B2M** |
| --- | --- | --- |
| Forward (5’-3’) | GAGCGATGGTGAGAGCTAAGGT | TCTCTCTCCATTCTTCAGTAAGTCAACT |
| Reverse (5’-3’) | CCCTAAAACCCGCCACATCT | CCAGCAGAGAATGGAAAGTCAA |
| Probe (5’-3’) | 5HEX/CCATCACCC/ZEN/  TCTACATCACCGCCC/3IABKFQ | 6-FAM/ATGTGTCTG/ZEN/  GGTTTCATCCATCCGACA/3IABKFQ |

*Definition of abbreviations:* B2M = beta-2-microglobulin; ND1 = NADH:ubiquinone oxidoreductase core subunit 1.

**SUPPLEMENTARY FIGURES**

Eosinophil count

Participants in ECLIPSE study

(*n* = 3,186)

Plasma samples not analyzed (n = 484)

Plasma samples analyzed

(*n* = 2,702)

COPD

(*n* = 2,128)

Current smokers without airway obstruction (*n* = 331)

Non-smokers

(*n* = 243)

GOLD stage

GOLD I/II

(*n* = 944)

GOLD III

(*n* = 893)

GOLD IV

(*n* = 290)

<300 cells/µL

(*n* = 1,640)

≥300 cells/µL

(*n* = 430)

**Supplementary Figure S1. Distribution of ECLIPSE study population groups.**

COPD = chronic obstructive pulmonary disease; ECLIPSE = Evaluation of COPD Longitudinally to Identify Predictive Surrogate Endpoints; GOLD = Global Initiative for Obstructive Lung Disease.

**SUPPLEMENTARY REFERENCES**

1. Agusti A, Calverley PMA, Celli B, Coxson HO, Edwards LD, Lomas DA, MacNee W, Miller BE, Rennard S, Silverman EK, Tal-Singer R, Wouters E, Yates JC, Vestbo J, Evaluation of COPD Longitudinally to Identify Predictive Surrogate Endpoints (ECLIPSE) investigators. Characterisation of COPD heterogeneity in the ECLIPSE cohort. *Respir. Res.* 2010; 11: 122.

2. Williams MC, Murchison JT, Edwards LD, Agustí A, Bakke P, Calverley PMA, Celli B, Coxson HO, Crim C, Lomas DA, Miller BE, Rennard S, Silverman EK, Tal-Singer R, Vestbo J, Wouters E, Yates JC, van Beek EJR, Newby DE, MacNee W, Evaluation of COPD Longitudinally to Identify Predictive Surrogate Endpoints (ECLIPSE) investigators. Coronary artery calcification is increased in patients with COPD and associated with increased morbidity and mortality. *Thorax* 2014; 69: 718–723.

3. Ware SA, Desai N, Lopez M, Leach D, Zhang Y, Giordano L, Nouraie M, Picard M, Kaufman BA. An automated, high-throughput methodology optimized for quantitative cell-free mitochondrial and nuclear DNA isolation from plasma. *J. Biol. Chem.* 2020; 295: 15677–15691.

4. Trumpff C, Marsland AL, Basualto-Alarcón C, Martin JL, Carroll JE, Sturm G, Vincent AE, Mosharov EV, Gu Z, Kaufman BA, Picard M. Acute psychological stress increases serum circulating cell-free mitochondrial DNA. *Psychoneuroendocrinology* 2019; 106: 268–276.

5. Belmonte FR, Martin JL, Frescura K, Damas J, Pereira F, Tarnopolsky MA, Kaufman BA. Digital PCR methods improve detection sensitivity and measurement precision of low abundance mtDNA deletions. *Sci. Rep.* 2016; 6: 25186.
